# Search strategies of Wikipedia readers

**DOI:** 10.1371/journal.pone.0170746

**Published:** 2017-02-02

**Authors:** Giovanna Chiara Rodi, Vittorio Loreto, Francesca Tria

**Affiliations:** 1 Polytechnic University of Turin, Department of Mathematical Sciences, Turin, Italy; 2 ISI Foundation, Turin, Italy; 3 Sapienza University of Rome, Physics Department, Rome, Italy; University of Warwick, UNITED KINGDOM

## Abstract

The quest for information is one of the most common activity of human beings. Despite the the impressive progress of search engines, not to miss the needed piece of information could be still very tough, as well as to acquire specific competences and knowledge by shaping and following the proper learning paths. Indeed, the need to find sensible paths in information networks is one of the biggest challenges of our societies and, to effectively address it, it is important to investigate the strategies adopted by human users to cope with the cognitive bottleneck of finding their way in a growing sea of information. Here we focus on the case of Wikipedia and investigate a recently released dataset about users’ click on the English Wikipedia, namely the English Wikipedia Clickstream. We perform a semantically charged analysis to uncover the general patterns followed by information seekers in the multi-dimensional space of Wikipedia topics/categories. We discover the existence of well defined strategies in which users tend to start from very general, i.e., semantically broad, pages and progressively narrow down the scope of their navigation, while keeping a growing semantic coherence. This is unlike strategies associated to tasks with predefined search goals, namely the case of the Wikispeedia game. In this case users first move from the ‘particular’ to the ‘universal’ before focusing down again to the required target. The clear picture offered here represents a very important stepping stone towards a better design of information networks and recommendation strategies, as well as the construction of radically new learning paths.

## Introduction

The World Wide Web is nowadays the most common source of information. While surfing, we jump into facts and news and, driven by curiosity or need, we continuously move from page to page, until novel stimuli push us to start other paths. One of the most dramatic aspects of the information age is that we know so little about the effects of the exponentially increasing amounts of information on human brains and human behaviours [1, 2]. Human brains are evolving in much longer timescales and our information processing capacity has presumably an hard time in changing in response to the complex information environment. Perception is a complex process that filters out enormous amounts of data and flags potentially relevant information to allow the individual to navigate the world as function of her needs and the environment’s affordances [3–5]. Still the quest of information is unavoidably linked to all our activities, be it learning, working or playing. In this scenario understanding how we behave as information-seekers [6, 7], and if any common patterns exist in our behaviours, is one of the big challenges of our era, with a potentially large impact in the way in which information should be structured and the educational schemes should be deeply rethought.

In this framework, particularly valuable is the case of Wikipedia [8], more often considered as a primary source of well-established and reliable information. It represents a huge system of pieces of knowledge, continuously created, updated and modified by users, as well as the networked structure in which they are embedded. In this *knowledge space*, our walks from article to article can be effectively thought of as *learning* paths [9, 10], either oriented towards very specific pieces of information or moved by curiosity. Comprehending how we act in such a space could be key to design new and more effective learning strategies as well as to improve the actual systems and platforms designed for knowledge search.

To date, no data on the individual behaviours of Wikipedia readers are available. For this reason, previous studies on the topic were based on the analysis of traffic flows data gathered locally. This is the case of the work by Ratkiewicz et al. [11] in which it is investigated the use of Wikipedia looking at the Web requests outgoing by the Indiana University over some months of browsing activities. In other cases, games lying on Wikipedia were designed to obtain the paths followed by the players while navigating to fulfil the gaming task under some temporal or spatial constraints. *The Wiki Game* by Clemesha [12] and *Wikispeedia* [13, 14] are two notable examples. In Wikispeedia players were asked to navigate on a reduced version of Wikipedia to go from a given starting page to a given target page always hopping on Wikipedia pages. Based on the paths gathered, West et al. reported [15] about emerging common navigation strategies.

In this paper we address a similar question, namely whether regularities are observed in the way users surf Wikipedia, by tapping on the recent release of data about Wikipedia users. Gathered during February 2015, the English Wikipedia Clickstream (EWC) [16], provide a large set of referer/resource pairs recurring in the Wikipedia logs more than 10 times during February 2015. The resources are all the pages in the main namespace of the English Wikipedia, i.e., all the articles pages and the MainPage. From this dataset, the graph of the actual traffic flows streaming in and within Wikipedia can be considered. Though not direct data about the navigation histories of individual users are yet available, the EWC dataset allows for the construction of first-order Markov chains, where the transition probabilities are given by the traffic flows, to be considered as statistically legitimate proxies for the real paths navigated by the users [17]. From this perspective the English Wikipedia Clickstream represents a very important step forward to address the question of the navigation strategies of users in information networks.

In a very recent work on the EWC dataset, Lamprecht et al. [18] focused on how Wikipedia pages’ style can bias the navigation. Unlike Lamprecht et al. contribution, the analysis presented here aims at characterizing the emerging paths from a semantic point of view to identify possible recurrent meaningful patterns. To this end we introduce a vectorial representation in which each Wikipedia page is represented by a vector of features in a 13-dimensional space [17, 19] whose dimensions correspond to broad Wikipedia topics/main categories [20]. More in details, they are the following categories: *geography, health, history, humanities, literature, mathematics, nature, people, philosophy, reference works, science, society, technology*. These very general subjects are here treated as coordinates of a topical abstract space in which we study users’ paths. The vector coordinates for each page in this space are computed so that the weights are proportional to the semantic relatedness of each topic with the page’s parent categories.

Based on this semantic mapping of Wikipedia pages we perform a thorough analysis of users’ paths and, splitting the paths according to their lengths, strong regular patterns emerge in users’ navigation behaviours. First of all, the longer the walk, the longer the user navigates deeper and deeper levels of specificity. Regardless of the path’s length, the navigation strategy emerges as quite universal, with the very first page visited being more abstract and of high level, and used by the reader to access her content of interest, typically more specific and concrete. The semantic distances spanned by the readers are not uniformly distributed along the jumps. The reader tends to do the longest semantic jumps at the beginning and towards the conclusion of her information exploration. In doing this the semantic coherence of her visits keeps increasing throughout the path.

Finally we contrasted the strategies displayed in the EWC dataset, where no goal for users is predefined, to the goal-oriented walks of Wikispeedia players. Here very different patterns and strategies are observed when rescaling the results according to the path lengths. In particular, users simulated to the English Wikipedia Clickstream typically enters the encyclopedia via more abstract and more interdisciplinary pages while in the next steps they tend to progressively narrow the focus of their visits. On the other hand, Wikispeedia’s players, for whom the starting and the target pages are randomly assigned by the system, move initially towards more abstract articles to find her way through the hyperlinks towards the target article. This strategy is independent of the path lengths and was already observed by West et al. [15].

## Materials and Methods

### The English Wikipedia Clickstream (EWC) dataset

The main dataset we considered in our analysis is the English Wikipedia Clickstream [16], released on February 2015. The dataset includes 22 millions of aggregated requests of articles in the main namespace of the English Wikipedia, together with their *referers*, i.e., the webpages from which the requests were performed by the users during the month of February 2015, and the number of occurrences of each pairs, if exceeding 10 requests. More in details, the data were extracted from the request logs of Wikipedia and aggregated so that the referer can correspond to an article or to an external source. In the first case, the article title is given, whereas if the page is not an article, it is explicitly reported the proper categories among the following: *google*, *twitter*, *bing*, *yahoo*, *facebook*, *wikipedia* (any page in Wikipedia different from an article), *internal* (any page belonging to a different internal Wikimedia project), an *empty* referer, or *other* for any different referer.

For our purposes, we cleaned the dataset by removing all the requests to non existing articles (e.g., articles requested by following any *redlink*, where a red link represents a link to a page that is either non-existent or deleted). All the other {referer, resource} pairs were kept, regardless of the fact that an hyperlink exists directly connecting the two articles. We furthermore chose to treat the MainPage of Wikipedia (in the dataset considered as an article page since it appears in the main namespace) as an external source of navigation, thus adding it to the set of external sources previously listed (in the following: *mainpage*). The choice was due to the fact the the MainPage is often used as a starting point for a research or navigation in the online encyclopedia similarly to any external web search engine, and it not semantically representable as we did for the other articles (and as it is described in the next paragraph).

**Fig 1:**
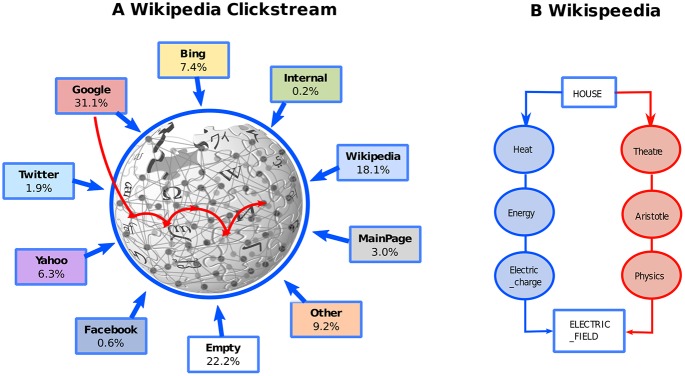
Datasets under consideration. In (A) we illustrate the English Wikipedia Clickstream dataset. The 9 different external sources plus the MainPage are illustrated with the fraction of flux outgoing from them. The paths we considered in our analysis start from one of the 9 sources to randomly walking over the Wikipedia articles accordingly to the transition counts provided by the dataset. (B) Two examples of paths followed by players of the Wikispeedia game, whose task was that of navigating on a reduced version of Wikipedia from a given starting page to a given target one (from *House* to *Electric_Field* in the example).

After cleaning, we ended up with a set of 3,087,211 articles. The requested {article, article} pairs were 14,076,289. Among these, the pairs of pages connected by a hyperlink are around 88%. The requested {external-source, article} pairs were 8,231,312. An illustration of the different fluxes coming from the external sources is reported in Fig 1(A).

### From the EWC dataset to Random Walks on Wikipedia

The EWC dataset provides the transition counts between pairs of Wikipedia pages. This information can be used to drive a random walker, whose paths over the Wikipedia pages will be here considered as proxies for the real behaviours of users who navigate Wikipedia. In doing so, we are assuming that the navigation over the encyclopedia can be represented by a stochastic process without memory. Indeed, while it was proven [17] that the human navigation processes are better modeled by second (or third) order Markov chains in topical and abstract level of description, still memoryless model are statistically legitimate to simulate human navigation on a page level, as done in the present paper.

To take full advantage of the available dataset, we refer to the traffic flowing into Wikipedia from the external sources to select the starting pages of the simulated walks. On the other hand, every page can be the last one of the walk with a probability proportional to the net difference between the incoming (from other pages and from external sources) and outgoing (only towards other wikipedia articles) traffic flux on that page.

More in details, we construct the Random Walks on Wikipedia based on the EWC dataset as follows. Each of the different external sources listed in the previous paragraph is in turn selected as the external origin of the simulated walker. This is the starting point of the walk.

- The first node after the origin is randomly selected among the ones reached by the source, with a probability proportional to the incoming flux;
- on every node encountered across the walk two complementary events can occur: either the walker takes one more step or the walk stops. The stopping probability at node *i* is defined as:
  $$\begin{array}{c} prob_{stop}^{i}=\max(0,1-\frac{s_{out}^{int}\left(i\right)}{s_{in}^{int}\left(i\right)+s_{in}^{ext}\left(i\right)}). \end{array}$$ (1)
  In the above definitions, $s_{in}^{ext}\left(i\right)$ and $s_{in}^{int}\left(i\right)$ are the incoming strength on node *i* coming, respectively, from all the external and internal source (with $s_{in}\left(i\right)=s_{in}^{int}\left(i\right)+s_{in}^{ext}\left(i\right)$) and $s_{out}^{int}\left(i\right)$ is the outgoing (i.e., towards other pages) strength of node *i*. In all the mentioned cases, the strength of node *i* with respect to a set of pages/sources *S* is here defined as *s*_*in*(*out*)_(*i*) = ∑_*j*∈*S*_ *w*_*ij*_, where *w*_*ij*_ is the counts of transitions from(to) j to(from) i;
- when the walk does not stop on a node, the next node is selected among its neighbours with a probability proportional to the transition counts.

With the above defined procedure, we simulated 10^7^ paths, originated from each of the different external sources. The average path length is around 1.4-1.5 nodes for all the sources, with the only exception of the MainPage for which it is slightly higher (around 1.8 nodes). The length distribution is reported in Fig A in S1 File.

### Wikispeedia game

In this paper we constrast the artificial paths generated through the EWC dataset with the true paths followed by players of the Wikispeedia game. Wikispeedia [14] was indeed designed to collect data about the strategies of humans while navigating a reduced version of Wikipedia to reach a known *target* page from a *starting* one, both randomly assigned by the system. The task was that of finding the shortest path connecting the two articles, only following hyperlinks shown in each visited page. The data gathered are available online and contain the entire tracks of the paths followed by the players. In addition, it is specified whether the game session was successful, i.e., whether the player managed to complete the task, namely to reach the preassigned goal, or, in the unsuccessful case, she gave up or encountered timeout. In the following, we will mainly refer to the subset of around 50,000 successfully paths. An example of a Wikispeedia task with two different successful realizations is reported in Fig 1(B).

### Semantic mapping of Wikipedia pages

In order to identify whether regular patterns exist in the way the information seekers navigate Wikipedia we need to abstract from the microscopic page level to a coarse-grained, semantically meaningful, representation. The problem of extracting a meaningful semantic mapping of Wikipedia pages is frequent in the literature. Many authors considered the pages content to derive a vector representation via Natural Language Processing Analysis, e.g., tfidf, short for term frequency-inverse document frequency, or word count analysis. This is the case of the already cited works by West et al. [15] and Ratkiewicz et al. [11]. In other studies the page content is not taken into account and one focuses instead on Wikipedia category structure. In particular, some top-level categories can be considered as main topical concepts, suitable for a semantic characterization of the pages. Here, following previous studies [17, 19, 21], we consider the top-level subcategories of the *Main_Topic_Classification* [20] container category as coordinates of a novel reduced space. Unlike Singer et al. method [17], we do not reduce the semantic complexity of each page to just one representative topic (in their work, the one from which the shortest-path to the page is the minimal). We assign instead to each page a topic distribution, as in [19], thus mapping the article into a point of the semantic space with 13 dimensions. These 13 topics/main categories are very general subjects and are here treated as coordinates of a topical abstract space where all the pages and users’ paths are studied. In the following we refer to them simply with *topic*s. Each article of Wikipedia is then mapped into a point of this 13-dimensional space, its corresponding semantic vector being computed so that the weights are proportional to the semantic relatedness of each topic with the article’s parent categories (see next paragraph). Once obtained the vectorial representation of each page, we consider some common measures in euclidean spaces, such as norms and similarities measures, to give a semantic interpretation of the position each page occupies along as their inter-relations. With these tools, the simulated paths can be read in the topical space and contrasted to some real paths, as the ones gathered with the Wikispeedia game.

#### Extraction of a vector representation

**Fig 2:**
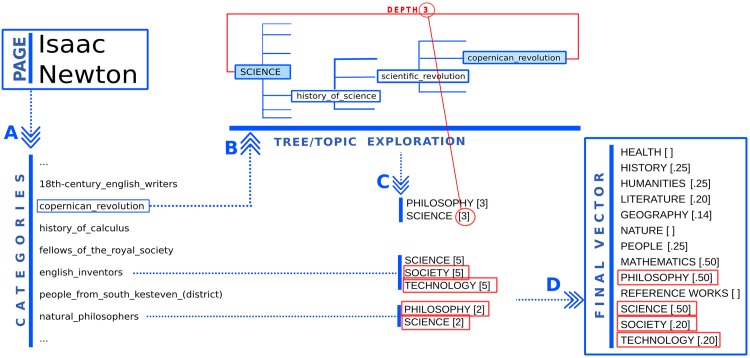
Example illustrating the construction of the topical vector for the Isaac Newton article. For the Isaac Newton page one first considers the list of parents categories (panel A). For each category, one identifies the most-representative-topics (panel B), selecting the ones from which the depth of the category in the categories tree is minimal. For each page, we consider the whole list of most-representative-topics and corresponding depths (panel C). For instance the category *copernican_revolution* has the smallest depth (equal to 3) in the tree of the topic *SCIENCE*. The vector representation of the coordinates of the main topics is now obtained by weighting each topic with the inverse of the minimal depth computed above (panel D). For instance the topic *SCIENCE* appears in the topical vector with weight 1/2.

The Wikipedia category system has a pseudo-hierarchical structure, where each page and category can have multiple parent categories. This fact, along with the lack of any central root from which the structure starts branching, turns into the possibility to always find a path along the categories structure to connect any category pairs. In particular this is true for the main topics categories listed in the previous paragraph. Indeed, if we consider the category tree rooted in each of the topics, every other category can be reached via breadth-first search starting from the roots. It follows that the depth at which the category is firstly encountered in a tree, is meaningful of the relevance of the rooting topic in charactering that category. Indeed, the lower the depth, the closer the category to the topic at the root, the higher the topic semantic relevance. Thus, by computing the smallest depth of each of the trees rooted in the 13 main topics, we can assign to each category the mostly relevant topics, namely the ones from which the depth are minimal. The procedure followed is illustrated in Fig 2 and now explained in details.

For any page, we first find the list of parent categories which the page belongs to (panel A of Fig 2). We assign to each parent category the set of the most-representative-topics. They are selected because are the ones, among the 13, from which the category depth is minimal (Fig 2, panel B). In this way, we obtain for each page a set of the most-relevant-topics and their corresponding-depths (panel C). From this set, the final vector representation is easily derived by computing the weight of each topic as the inverse of the minimal depth found for it (panel D). We choose to consider the inverse so that the weights were proportional to the semantic values, and the most-representative-topics would mostly contribute in the evaluation of typical vector measures like the norm.

All the data about the category system were extracted from the Wikipedia category links dump [22], accessed on date 10-22-2015. In the Supporting Information, we demonstrate the robustness of the procedure by reporting results corresponding to another dump (obtained on 04-03-2015) and to a slight modified procedure to construct the topical vectors. While generating the trees rooted in the topics, the maintenance categories were ignored, such as tracking and hidden categories. Specifically, we did not consider the following categories and their direct subcategories: *wikipedia categorization*, *hidden categories*, *tracking categories*, *disambiguation categories*, *namespace example pages*. For some pages no vector representation was derived, since at the time of the dump, they belonged only to some maintenance categories. They were about 5% of the total number of pages appearing in the EWC dataset and they were excluded from the successive analysis.

#### Observables used for analysis

To characterize the Wikipedia articles in the reduced semantic representation, we refer to two main measures: the norm and the entropy of the corresponding topical vectors.

- The **vector norm** is the usual L2 norm, normalized to the square root of the space dimension, i.e., the number of topics. For a generic page A, whose vector *w*_*A*_ has components $w_{A}^{t}$ for the different topic *t* ∈ [1, *T*], with *T* = 13:
  $$\begin{array}{c} ∥w_{A}∥=\sqrt{\frac{1}{T}\sum_{t=1}^{T}{\left(w_{A}^{t}\right)}^{2}} \end{array}$$ (2)
  With this choice, the norm is always in the (0 : 1] range, with higher values corresponding to pages with more abstract content and lower values to more specialized pages.
- In order to measure the level of multidisciplinarity of a vector, we compute the **entropy** *S*(*w*_*A*_) of vector *w*_*A*_ as:
  $$\begin{array}{c} S\left(w_{A}\right)=-\frac{1}{log_{2}\left(T\right)}\sum_{t=1}^{T}{\hat{w}}_{A}^{t}\log_{2}\left({\hat{w}}_{A}^{t}\right) \end{array}$$ (3)
  with ${\hat{w}}_{A}^{t}=w_{A}/\sum_{t=1}^{T}w_{A}^{t}$, so that the weights sum to 1. High values in entropy means a very general or multidisciplinary content, while the low-entropy pages are pages semantically connected with only one knowledge field.

**Fig 3:**
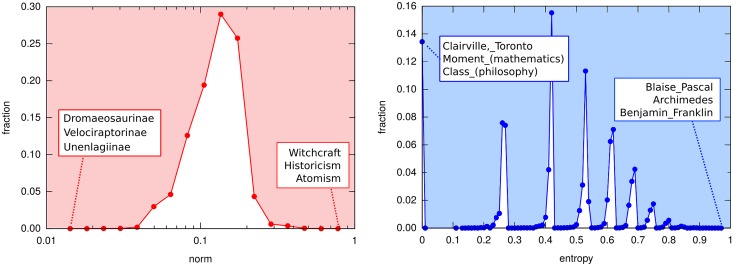
Distributions of page norms (left) and entropies (right). The distributions are computed over the set of all pages for which a vector representation was derived. For both norm and entropy, in the boxes some exemplar pages are reported to illustrate the meaning of extreme values.

Fig 3 reports the distributions of the norms and entropies for all the pages considered as well as some example of pages lying at the extremes of the distributions. It is worth noting the particular shape of the resulting entropy distribution, reported in the subfigure on the right. Indeed, the topical vector entropies tend to distribute in isolated spikes. This is due to the input points which belong to a discrete space.

In addition to the observables introduced above, we considered also distances and similarities between vectors:

- **distance** *d*(*w*_*A*_, *w*_*B*_)—It is the usual L2 distance between the vectors *w*_*A*_ and *w*_*B*_:
  $$\begin{array}{c} d\left(w_{A},w_{B}\right)=\sqrt{\frac{1}{T}\sum_{t=1}^{T}{\left(w_{A}^{t}-w_{B}^{t}\right)}^{2}}; \end{array}$$ (4)
- **similarity** *sim*(*w*_*A*_, *w*_*B*_)—It is the cosine similarity of the two vectors *w*_*A*_ e *w*_*B*_:
  $$\begin{array}{c} sim\left(w_{A},w_{B}\right)=\frac{1}{T}\frac{w_{A}\cdot w_{B}}{∥w_{A}∥∥w_{B}∥} \end{array}$$ (5)

These two quantities give complementary information about how close two pages are in the semantic space. Indeed, while the distance gives us an overall idea of how far two pages are in terms of both content diversity and depth, the similarity is more directly related to the extent of their semantic overlap, i.e., regardless of the difference in depths.

## Results

We here report results obtained by means of the semantic measures defined in the previous section on our artificially generated paths. We start by splitting the walks according to their lengths *l*. On each subset of walks of give length, we considered all the nodes encountered, gathered according to their position *k* along the path counted from the end. In this way, we replicate the same alignment proposed by West et al. in their work [15], thus assuming that the node where the navigation ends is the target node of the user surfing the encyclopedia. With this choice, in terms of notation, the first nodes encountered have index *k* = *l*, while the last ones have *k* = 0. We also set $w_{k}^{l}$ to represent the vector of a node encountered *k* steps before the end on a path of length *l*.

At a first level of analysis, we evaluated the above defined observables by averaging over all the nodes appearing at position *k* of the walks, for fixed path lengths. In particular we computed: (A) the average norm $\bar{∥w_{k}^{l}∥}$, (B) the average entropy $\bar{S(w_{k}^{l})}$, (C) the average distance $\bar{d(w_{k}^{l},w_{k-1}^{l})}$ and (E) similarity $\bar{sim(w_{k}^{l},w_{k-1}^{l})}$ between each node and the next visited along the path, (D) the average distance and (F) similarity between each node and the last one in the corresponding path, respectively $\bar{d(w_{k}^{l},w_{0}^{l})}$ and $\bar{sim(w_{k}^{l},w_{0}^{l})}$.

**Fig 4:**
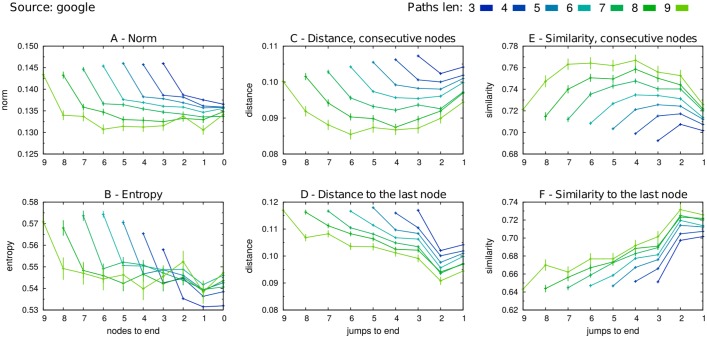
Paths generated from the external source *google*: averages. The 10^7^ paths simulated with *google* as source were split by lengths. For each fixed length *l*, we computed the averages of the following quantities over all the nodes(pairs) at *k* steps(jumps) to the end: (A) the average norm $\bar{∥w_{k}^{l}∥}$, (B) the entropy $\bar{S(w_{k}^{l})}$, (C) the distance and (E) the similarity between all the pairs of nodes consecutively visited along each path, respectively $\bar{d(w_{k}^{l},w_{k-1}^{l})}$ and $\bar{sim(w_{k}^{l},w_{k-1}^{l})}$, (D) the distance and (F) the similarity between every node visited and the ending node along each path, i.e. $\bar{d(w_{k}^{l},w_{0}^{l})}$ and $\bar{sim(w_{k}^{l},w_{0}^{l})}$. The error bars display the standard errors of the means. Each color refers to a path length, from 3 (blue) to 9 (light green).

In Fig 4 we present the results obtained on simulated walks where *google* was chosen as the external source to weight the starting probabilities. In this figure, as well as in the following ones, we align the last nodes of the paths (i.e. position *k* = 0) to the right.

Regular patterns across paths of all lengths in the trends of the six observables can be clearly observed. From the norm subfigure (A), it emerges that with the first step, whatever the length of the walk, the simulated walker moves from quite general and abstract pages to more specific ones, further slightly increasing the specificity of the pages while pushing the walk. Complementary, the analysis of the entropy (B) tell us that the simulated Wikipedia reader typically access the encyclopedia from *google* via interdisciplinary articles, focusing on more defined field in the very first steps of her navigational path.

From the analysis of the pairs measures, it appears that the reader tends to span a bigger space—both in terms of distance (C) and similarity (E)—at the beginning and ending of her walks, the steps in the middle still contributing to get her closer to the node where she stops the navigation. Quite interestingly, patterns very similar to the ones emerging in the semantic distance between consecutive nodes, subfigure (D) in Fig 4 are found by Mastroianni et al. in a recent contribution [23], while investigating via GPS tracking the average spacial distance travelled by single vehicles. In their work, they found a rescaling law of the patterns, which turns into the emergence of a universal behaviour of the drivers, independent of the path length, with the longer distances between successive stops appearing—as in our case—at the beginning and ending of the paths.

**Fig 5:**
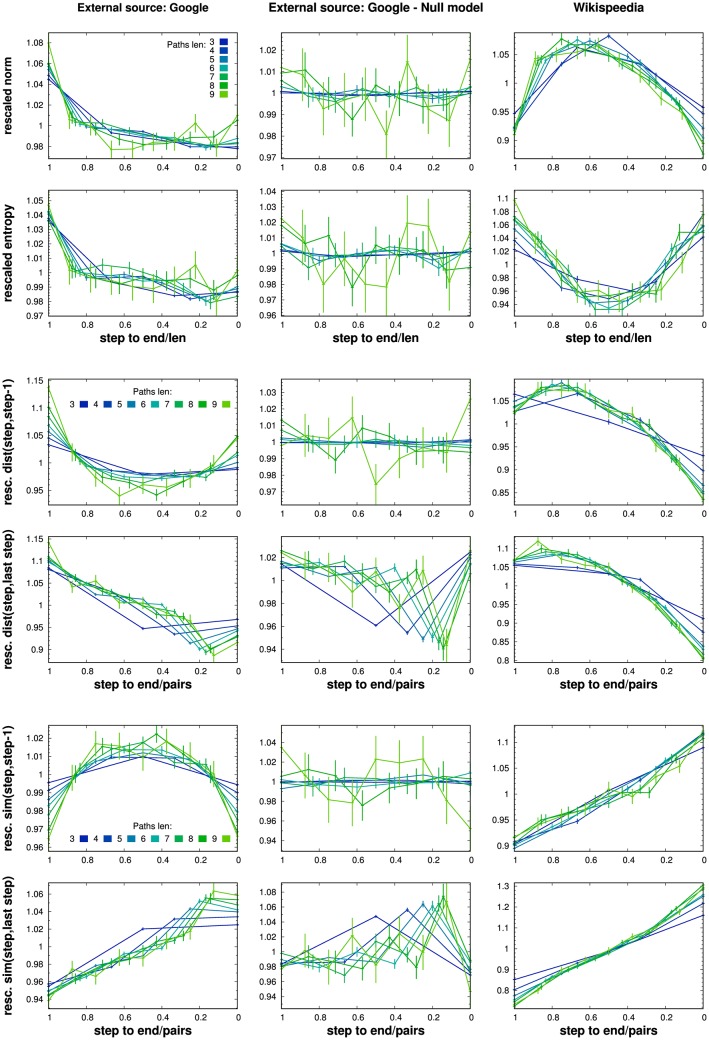
Rescaled averages over the simulated paths. In this panel we report the same data of Fig 4 (left column) after rescaling. The walks lengths are normalized to 1. The corresponding averages for step of the different measures (A)-(F) are rescaled with the mean value of the same measures evaluated over the whole set of nodes belonging to paths with the same length. The averages used to rescale the data are displayed in Fig D in S1 File. In the central and right columns similarly processed data are reported which refer respectively to a semantically uncorrelated model based on the *google* paths and to the Wikispeedia paths. Each color refers to a path length, from 3 (blue) to 9 (light green). The standard error of the means are reported.

In Fig 5 we report the same data as in Fig 4, suitably rescaled in order to highlight emerging universalities. In addition, we report the same results computed over two different datasets, in order to contrast the data. With *null model* we refer to 10^7^ paths still generated using *google* as external source, but after renaming the nodes, i.e. reassigning randomly the semantic vector representations to the whole set of pages. By doing this reassignment, we destroy any semantic correlation between the pages, while preserving the topology of the transition counts graph. The second dataset considered to contrast our data is the dataset of the paths gamed in Wikispeedia [14].

In all the plots, the x-axis is rescaled in such a way that all the paths show unitary length, while the averages reported in the y-axis are now further normalized with the average of the considered observable over all the nodes in the paths of corresponding length. As an example, the rescaled average norm reads $\bar{∥w_{k}^{l}∥}/<\bar{∥w_{k}^{l}∥}{>}_{k}$, with $<\bar{∥w_{k}^{l}∥}{>}_{k}=\frac{1}{k}\sum_{k}\bar{∥w_{k}^{l}∥}$, and similarly for the other observables. The global averages: (A) the average norm $<\bar{∥w_{k}^{l}∥}{>}_{k}$, (B) the entropy $<\bar{S(w_{k}^{l})}{>}_{k}$, (C) the distance and (E) similarity between consecutive nodes, respectively $<\bar{d(w_{k}^{l},w_{k-1}^{l})}{>}_{k}$ and $<\bar{sim(w_{k}^{l},w_{k-1}^{l})}{>}_{k}$, and finally (D) the average distance and (F) similarity to the last node of each path, respectively $<\bar{d(w_{k}^{l},w_{0}^{l})}{>}_{k}$ and $<\bar{sim(w_{k}^{l},w_{0}^{l})}{>}_{k}$, are reported in Fig D in S1 File. With such a rescale, in Fig 5 we find an interesting overlaps of the curves of different length. This points to a kind of universal behaviour emerging in searching strategies, independently of the length of the search.

First we note that after breaking the semantic correlations between the pages visited (in the *null model*), any regular pattern in the quantities observed disappears, as can be seen by comparing the figure in the left column with the central ones. It is worth noting a regularity which is not destroyed in the null model, related to the presence of loops in which users jump from the last visited page to the previous visited one, before definitively ending in the last. In those cases, both in the actual paths and in the reshuffled ones, the distance and similarity between the third to last and the last nodes (which coincide) are respectively zero and 1, reflecting in the odd behaviour of the point before the last in graphs (D) and (F), central column (those cases occur in our simulations with frequency within 0.05 (for 3 jumps walk) and 0.07 (for length 9)).

In both the remaining cases, *google* and Wikispeedia, the curves referring to different path lengths collapse into diverse and defined patterns, thus differently characterizing the strategies followed by the users/gamers. The reader who accesses Wikipedia from *google*, enters the encyclopedia via pages more abstract and more interdisciplinary than the ones she will click on in the following steps. On Wikispeedia, where both the starting page and the target are randomly assigned by the system, the player needs to move towards most abstract articles to find her way through the hyperlinks towards the target article. This strategy is independent of the path lengths and was already observed by West et al. [15], after analysing a bunch of heterogeneous measures over the same paths. Interestingly, while the starting and final pages are quite general, the article the player goes through to connect them are more specific to narrower fields of knowledge.

Also if we look at the pairs quantities (distance and similarity), the different strategies of the information-seeker users of Wikipedia and of the goal-oriented players of Wikispeedia emerge clearly. The first steps are used by the player to make big semantic jumps, the distance to the known target starting diminishing only after some steps. From that moment on, the player gets closer and closer to the target. Still, the similarity between successive pages and between each node and the target increases monotonically. This means that every step is used to enlarge the semantic overlapping, for example in terms of common fields of knowledge supporting the corresponding vectors.

The reader of Wikipedia, instead, uses the first page to direct her navigation: the first jump is always the one connecting the most distant pages. Then the following jumps are smaller and much similar, until the last one, significantly longer than the previous ones. A similar, but reversed, behaviour emerges for the similarity.

When sources different than *google* are considered, very similar trends emerge. We report the results obtained for the unrescaled measures in Figs E-M in S1 File. Only the entropy has a much more flat trend when the sources are the *mainpage*, *facebook* and *empty*. Furthermore, and more interestingly, if the reader accesses Wikipedia directly browsing the page name (i.e. in the *empty* referer case), the starting jump in norm is missed, as she went straight to the page of interest (Fig M(A) of S1 File).

**Fig 6:**
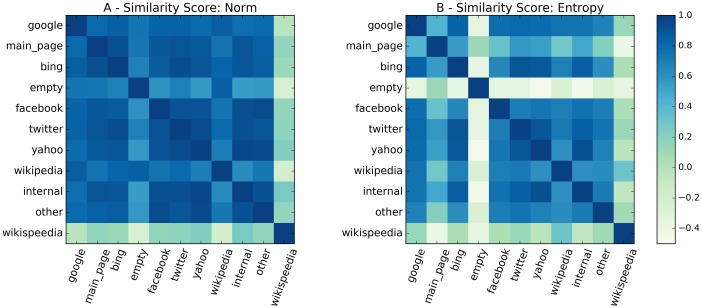
Similarity scores between sources. For the two observables norm (left panel) and entropy (right panel), we report the matrix of similarities score between all the sources and Wikispeedia. The score is defined by Eq (6). For each pair of sources, the unrescaled averages values of the observable are considered (as in Fig 4). Then, for each path length between 4 and 9, the Spearman correlation coefficient is computed between the averaged values of the observable. The final score is the obtained after averaging over all the lengths.

Finally, the differences in the patterns found depending on the walk source (or task for Wikispeedia) are synthetically depicted in Fig 6.

There, for the two observables norm and entropy (left and right subplot respectively), we report a similarity score computed as follows. For any pair of sources, say *A* and *B* and for any length *l* between 4 and 9, we have computed the Spearman coefficient of the average quantity along the *l* steps. Then, the coefficients found have been averaged over the different lengths. For the generic observable *q*, the similarity score between the sources is thus defined as:

$$\begin{array}{c} sim\_score{\left(A,B\right)}_{q}={\left\langle spear\left(\bar{q(w_{A}^{l})},\bar{q(w_{B}^{l})}\right)\right\rangle }_{l}. \end{array}$$ (6)

With this definition similarities in the norm and entropy trends along any walk are outlined by the score, as shown in Fig 6, aggregating results for different path lengths. Indeed, the Wikispeedia case stands out clearly as very uncorrelated (or even negatively correlated) to all the other sources. Contrasting the EWC sources only, the entropy maps allows to confirm the qualitative observations done in the Supplementary Information about the unlike behaviour of *empty* and *main_page* sources. In particular for the former, the dissimilarity with respect to all the other sources is even sharper than for the Wikispeedia case.

## Discussion

In the present work, we investigated the navigation behaviours of Wikipedia readers. Though no real navigation paths are available, we exploited the English Wikipedia Clickstream (EWC) dataset [16], gathered by Wikipedia during February 2015, that provides the transition probabilities between pairs of Wikipedia pages. EWC represents a very good proxy for real paths navigated by users [17]. Based on the actual transition probabilities EWC provides, we simulated realistic navigation paths through a memoryless markovian model.

The simulated paths were analysed on a semantic, abstract level, rather than on the microscopic page level. We took advantage of the Wikipedia category system to map each page into a point of a topical space. We used different generalizations of the usual L2 metric to characterize the semantic profile of the articles in such space, e.g., norms, entropy, distances and similarities. These quantities are the observables we considered to quantify users’ strategies while navigating Wikipedia.

By contrasting the results with other navigation paths based on suitably devised null models and on the real navigation paths of players of the Wikispeedia game, we were able to reveal the existence of very clear and regular patterns in the users’ navigation behaviours. For instance, the longer the walk, the longer the user navigates deeper and deeper levels of specificity. Still, regardless of the length, the navigation strategy emerges as quite universal, with the very first page navigated being more abstract and of high level, and used by the reader to access her content of interest, typically more specific and concrete. As in real physical paths travelled by car drivers [23], the semantic distances spanned by the readers are not uniformly distributed along the jumps. Readers tend to perform the longest semantic jumps at the beginning and towards the end of their exploration. However, the semantic coherence keeps increasing throughout the paths. Moreover and in accordance with the expectations, the differences with Wikispeedia paths reveal that Wikipedia readers do not have a well defined target in mind, their task being no so goal-oriented as for Wikispeedia players.

In conclusion, some features emerged as universal of the simulated Wikipedia users’ navigation paths. Different lengths of the paths correspond to different levels of specificity of the corresponding information-seeking tasks. Furthermore and most intriguingly, the strategies are independent of the task difficulty: users goes from an abstract starting page to access more specific content, whatever the external source they come from. Despite the simple assumptions made to generate realistic navigation paths, still the hints provided by the results represent important indications of the strategies used by learners/information seekers while exploring well structured knowledge spaces, as Wikipedia. The emerging picture presented here provides, in our opinion, a very important stepping stone towards a better design of information networks and recommendation strategies, as well as the construction of radically new learning paths.

## Supporting Information

S1 File Supporting information file. (PDF)

## References

[1] Giedd JN, Chied MD. The Digital Revolution and Adolescent Brain Evolution. J Adolesc Health. 2012;51(2):101–105. 10.1016/j.jadohealth.2012.06.002 22824439 PMC3432415

[2] Levitin DJ. The Organized Mind: Thinking Straight in the Age of Information Overload. Dutton; 2014.

[3] Foerde K, Knowlton BJ, Poldrack RA. Modulation of competing memory systems by distraction. Proceedings of the National Academy of Sciences. 2006;103(31):11778–11783. Available from: http://www.pnas.org/content/103/31/11778.abstract 10.1073/pnas.0602659103 PMC1544246 16868087

[4] Just MA, Keller TA, Cynkar J. A decrease in brain activation associated with driving when listening to someone speak. Brain Res. 2008;1205:70–80. 10.1016/j.brainres.2007.12.075 18353285 PMC2713933

[5] Schweizer TA, Kan K, Hung Y, Tam F, Naglie G, Graham S. Brain activity during driving with distraction: an immersive fMRI study. Frontiers in Human Neuroscience. 2013;7(53). Available from: http://www.frontiersin.org/human_neuroscience/10.3389/fnhum.2013.00053/abstract 10.3389/fnhum.2013.00053 23450757 PMC3584251

[6] Navarro-Prieto R, Scaife M, Rogers Y. Cognitive strategies in web searching. In: Proceedings of the 5th Conference on Human Factors & the Web; 1999.

[7] Gwizdka J. Distribution of Cognitive Load in Web Search. J Am Soc Inf Sci Technol. 2010 11;61(11):2167–2187. Available from: 10.1002/asi.v61:11

[8] Wikipedia, The Free Encyclopedia;. Date of access: 2015-10-22. http://en.wikipedia.org/

[9] Novikoff TP, Kleinberg JM, Strogatz SH. Education of a model student. Proceedings of the National Academy of Sciences. 2012;109(6):1868–1873. Available from: http://www.pnas.org/content/109/6/1868.abstract 10.1073/pnas.1109863109 PMC3277570 22308334

[10] Rodi GC, Loreto V, Servedio VDP, Tria F. Optimal Learning Paths in Information Networks. Scientific Reports. 2015;10286. 10.1038/srep10286 PMC4450758 26030508

[11] Ratkiewicz J, Menczer F, Fortunato S, Flammini A, Vespignani A. Traffic in Social Media II: Modeling Bursty Popularity. 2010 IEEE Second International Conference on Social Computing. 2010 aug;p. 393–400.

[12] Clemesha, Alex. The Wiki Game;. http://thewikigame.com/

[13] Wikispeedia;. http://cs.mcgill.ca/~rwest/wikispeedia/

[14] West R, Pineau J, Precup D. Wikispeedia: An Online Game for Inferring Semantic Distances between Concepts. IJCAI. 2009;p. 1598–1603.

[15] West R, Leskovec J. Human wayfinding in information networks. Proceedings of the 21st international conference on World Wide Web - WWW’12. 2012;p. 619.

[16] Wulczyn E, Taraborelli D. Wikipedia Clickstream. 2015; Available from: https://figshare.com/articles/Wikipedia_Clickstream/1305770

[17] Singer P, Helic D, Taraghi B, Strohmaier M. Detecting memory and structure in human navigation patterns using Markov chain models of varying order. PLoS ONE. 2014;9(7). 10.1371/journal.pone.0102070 PMC4094564 25013937

[18] Lamprecht D, Helic D, Strohmaier M. Quo vadis? On the Effects of Wikipedia’s Policies on Navigation. In: ICWSM Workshop; 2015. p. 64–66.

[19] Kittur A, Chi EH, Suh B. What’s in Wikipedia? Mapping Topics and Conflict Using Socially Annotated Category Structure. Chi. 2009;p. 1509–1512.

[20] Wikipedia, Main Topic Classification;. Date of access: 2015-10-22. https://en.wikipedia.org/wiki/Category:Main_topic_classifications

[21] Suchecki K, Salah AAA, Gao C, Scharnorst A. Evolution of Wikipedia’s Category Structure. Advances in Complex Systems. 2012 6;15(supp01):1250068 10.1142/S0219525912500683

[22] Wikipedia, dumps;. Date of access: 2015-10-22. https://dumps.wikimedia.org

[23] Mastroianni P, Monechi B, Servedio VDP, Liberto C, Valenti G, Loreto V. Individual Mobility Patterns in Urban Environment. In: Proceedings of the 1st International Conference on Complex Information Systems (COMPLEXIS 2016); 2016. p. 81–88.

