# Supplementary material for: Search strategies of Wikipedia readers
Source: PLoS One. 2017 Feb 2;12(2):e0170746. doi: 10.1371/journal.pone.0170746 (PMC5289465; doi:10.1371/journal.pone.0170746)
Supplement: S1 File — (PDF) [file pone.0170746.s001.pdf]

Search Strategies of Wikipedia Readers
Supporting Information

Giovanna Chiara Rodi[1,2,◕,*], Vittorio Loreto [3,2,◕], Francesca Tria[3,2, ◕]

1 Polytechnic Univ. of Turin, Dept. of Mathematical Sciences, Corso Duca degli Abruzzi, 24, 10129, Turin, Italy
2 ISI Foundation, Via Alassio 11/c, 10126, Turin, Italy
3 Sapienza Univ. of Rome, Physics Dept., P.le A. Moro 2, 00185, Rome, Italy

◕These authors contributed equally to this work.
*** giovannachiara.rodi@polito.it**

Walks lengths distribution

In Fig. A, we report the distribution of the lengths of the simulated paths, originated from the available 10 sources classified in the dataset and described in the main text. From each source, we generated 10^7 paths.

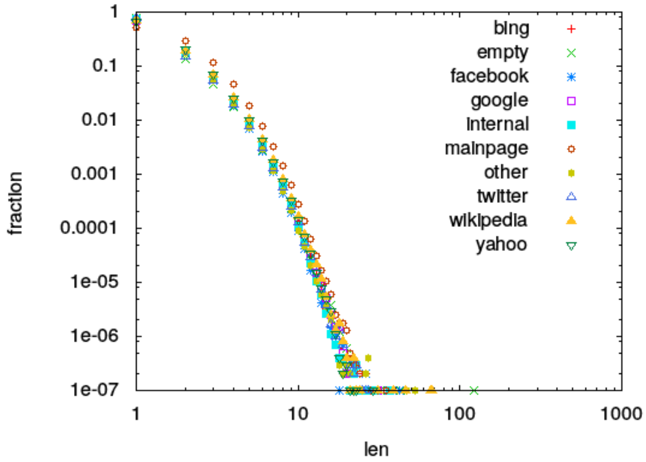

Figure A. Distribution of the path lengths. For the 10 different external sources, we report the distributions of the lengths of the simulated walks. From each source, we ran 10^7 simulations.

Semantic vectors representation: robustness results

The data and results reported in the main text are based on a dump of the English Wikipedia dated 10-22-2015. In this section, we report the same analysis on the paths originated from the source *google*, but referring to a different dump and a slightly modified procedure for the vector extraction.

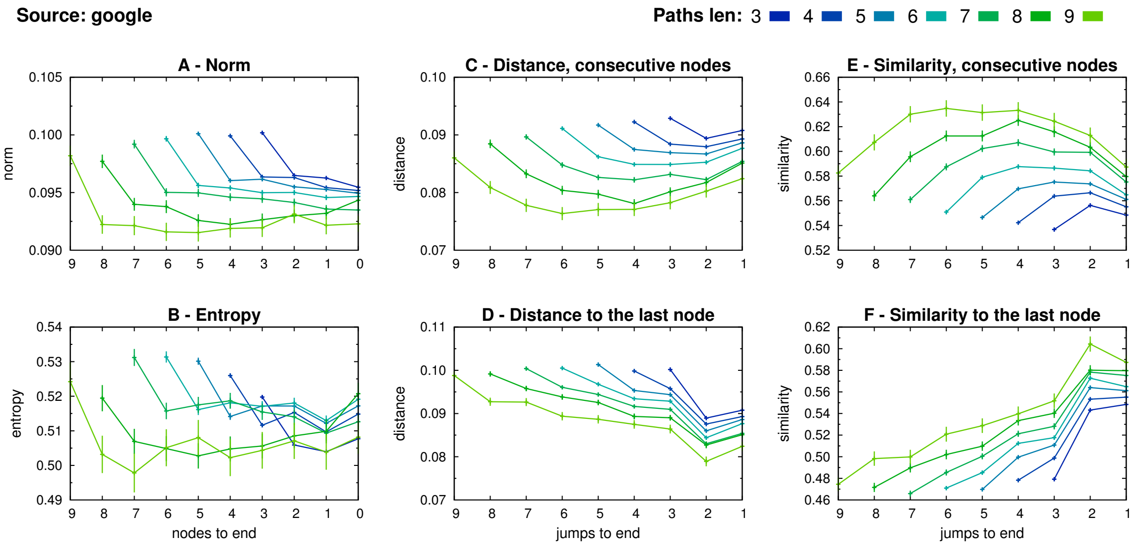

Figure B. Paths generated from the external source *google*: averages over a 38-topic semantic space. The 10^7 paths simulated with *google* as source were split by lengths. For each fixed length l, we computed the averages of the following quantities over all the nodes(pairs) at k steps(jumps) to the end: (A) the average norm $\overline{\|w_k^l\|}$, (B) the entropy $\overline{S(w_k^l)}$, (C) the distance and (E) the similarity between all the pairs of nodes consecutively visited along each path, respectively $\overline{d(w_k^l, w_{k-1}^l)}$ and $\overline{sim(w_k^l, w_{k-1}^l)}$, (D) the distance and (F) the similarity between every node visited and the ending node along each path, i.e. $\overline{d(w_k^l, w_0^l)}$ and $\overline{sim(w_k^l, w_0^l)}$. The error bars display the standard errors of the means. Each color refers to a path length, from 3 (blue) to 9 (light green).

The dump here considered is dated 04-03-2015. In it, the subcategories of the Main_Topic_Classification were 38, and namely: *agriculture, architecture, arts, chronology, creativity, culture, education, employment, energy, environment, geography, goods, government, health, history, humanities, humans, industry, information, knowledge, language, law, mathematics, medicine, mind, nature, objects, people, politics, science, sports, structure, systems, technology, telecommunications, universe, world.*

For each page, in extracting its vector representation based on the 38 coordinated listed above, we performed the first three phases as explained in the main text (Fig. 2 A-C): we selected the categories to which each page belongs to, and for each category we identified its most representative topic(s). This was the one(s), among the 38, from which the category depth is minimal. This depth is the semantic representativeness of the topic. In the original procedure, for each topic only the smallest depth over the categories was considered when deriving the final vector. Its inverse was chosen as corresponding weight. Here, instead of considering the smallest contribution, for each topic we average the depths over all the categories for which that topic is the most representative. The inverse of the average is the novel weight for the topic in the final vector.

With this choice of vector representation, we replicate the analysis of the paths originated from *google*. The results of the averages over each position along paths of fixed lengths, and of the same averages rescaled via the aggregated means are reported in Fig. B and Fig. C respectively.

We notice that the rescaled data and the trends reproduce what reported in the main text.

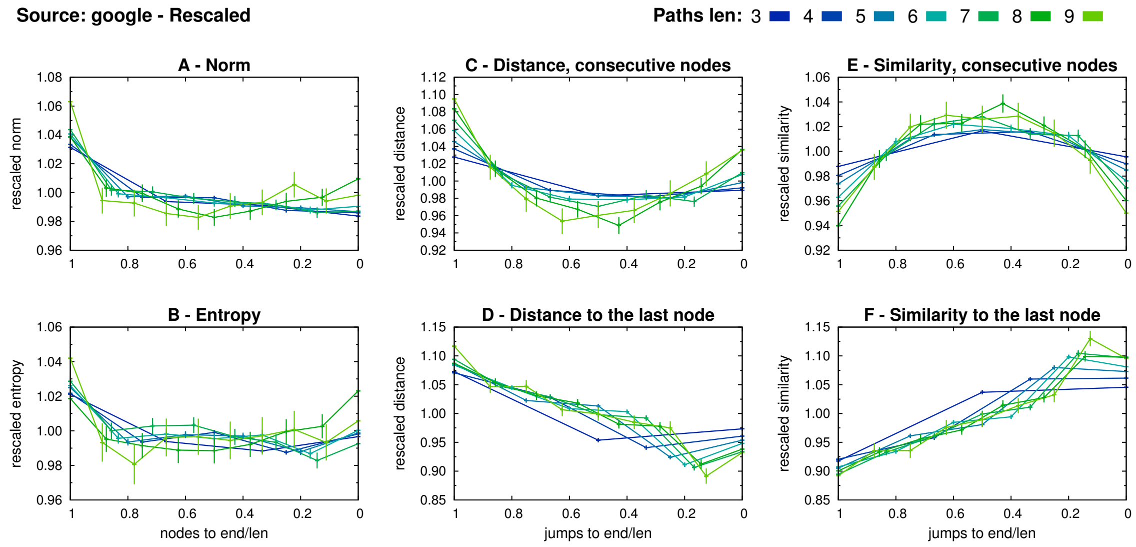

Figure C. Paths generated from the external source *google*: averages over a 38-topic semantic space. Rescaled. The data reported in Fig. B are here displayed after rescaling. The walks lengths are normalized to 1. The corresponding averages for step of the different measures (A)-(F) are rescaled with the mean value of the same measures evaluated over the whole set of nodes belonging to paths with the same length. Each color refers to a path length, from 3 (blue) to 9 (light green). The standard error of the means are reported.

Averages over unrescaled paths

In the following figure, the global averages of the different observables shown in Fig. 4 of the main text are reported for the three datasets(*google*, the *null model*, and Wikispeedia. The averages are: (A) the average norm $< \overline{\|w_k^l\|} >_k$, (B) the entropy $< \overline{S(w_k^l)} >_k$, (C) the distance and (E) similarity between consecutive nodes, respectively $< \overline{d(w_k^l, w_{k-1}^l)} >_k$ and $< \overline{sim(w_k^l, w_{k-1}^l)} >_k$, and finally (D) the average distance and (F) similarity to the last node of each path, respectively $< \overline{d(w_k^l, w_0^l)} >_k$ and $< \overline{sim(w_k^l, w_0^l)} >_k$.

As expected, no patterns emerge in the results for the *null model*. Diversely, the averages over *google* paths seems to follow a trend, e.g. the average norm or the distance between consecutive nodes decrease over paths of increasing length.

These averages are used in the main text to rescaled the observables trend along the paths, thus obtaining the results shown in Fig. 5.

Paths rescaled: miscellaneous sources

In this section we report the averages of the usual measures over paths simulated with different sources. As in the analysis reported in the main text, the semantic space we consider is again the one referring to the Dump dated 10-22-2015, i.e. with 13 topic-coordinates.

The sources here discussed are: *twitter*, *bing*, *yahoo*, *facebook*, *wikipedia* (any page in Wikipedia different from an article), *internal* (any page belonging to a different internal Wikimedia project), an *empty* referer, or *other* for any different referers. The Wikipedia incoming traffic fluxes from each of them are illustrated in Fig. 1 of the main text.

We can note that the trends of the quantities are quite similar to the ones emerging when *google* is the source (Fig. 4 of the main text). Still, we observe that the entropy is less

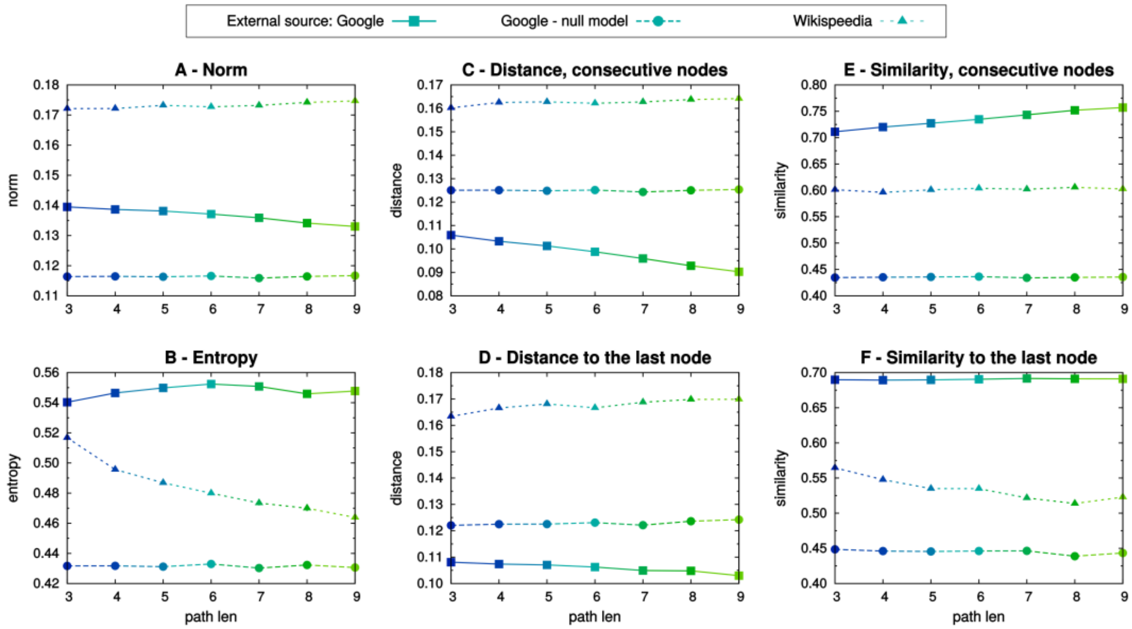

Figure D. Averages over the aggregated paths. The averages of measures introduced in Fig. 4 of the main text are here computed over all the nodes encountered along walks of fixed length l. Squares, circles and triangles refer respectively to the paths generated from the source *google*, to the same paths but semantically reshuffled (null model) and on the paths generated in the Wikispeedia game. Using the same palette of Fig. 4, each color refers to a length. The standard errors of the means are reported, though not visible at this scale.

informative when the sources are the *mainpage* Fig. F, *facebook* Fig. H and *empty* Fig. M. Moreover, in this last case the norm behaves differently too. Indeed, the large semantic jump typically seen at the first step of the walk is missed here, when the reader browse directly the first Wikipedia article, as she went straight to her content of interest (Fig. S12(A)).

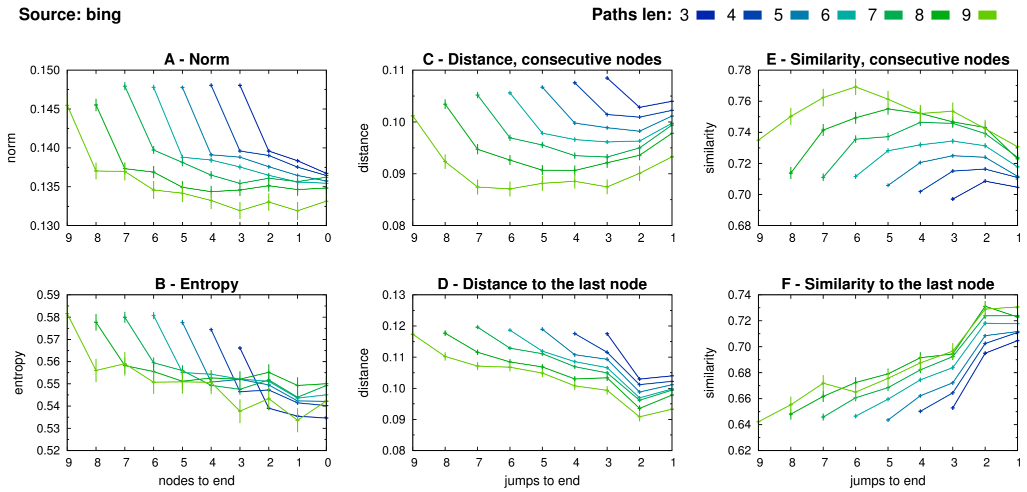

Figure E. Paths generated from the external source *bing*: averages. The 10^7 paths simulated with *bing* as source were split by lengths. For each fixed length l, we computed the averages of the following quantities over all the nodes(pairs) at k steps(jumps) to the end: (A) the average norm $\overline{\|w_k^l\|}$, (B) the entropy $\overline{S(w_k^l)}$, (C) the distance and (E) the similarity between all the pairs of nodes consecutively visited along each path, respectively $\overline{d(w_k^l, w_{k-1}^l)}$ and $\overline{sim(w_k^l, w_{k-1}^l)}$, (D) the distance and (F) the similarity between every node visited and the ending node along each path, i.e. $\overline{d(w_k^l, w_0^l)}$ and $\overline{sim(w_k^l, w_0^l)}$. The error bars display the standard errors of the means. Each color refers to a path length, from 3 (blue) to 9 (light green).

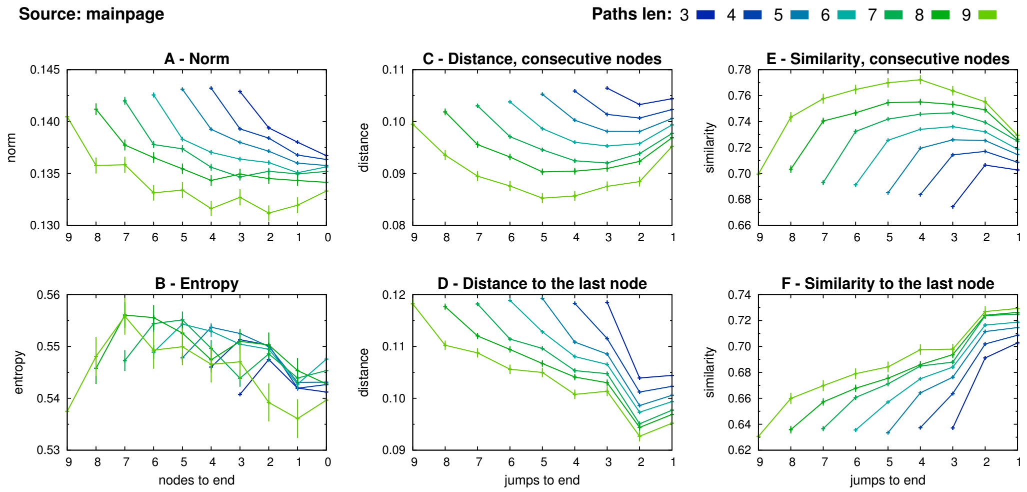

Figure F. Paths generated from the external source *main page*: averages. As in E.

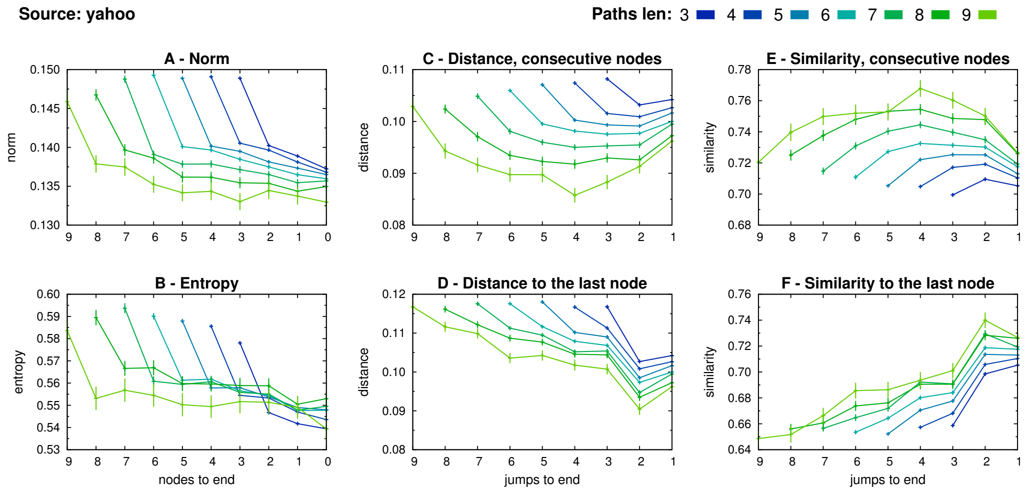

Figure G. Paths generated from the external source *yahoo*: averages. As in E.

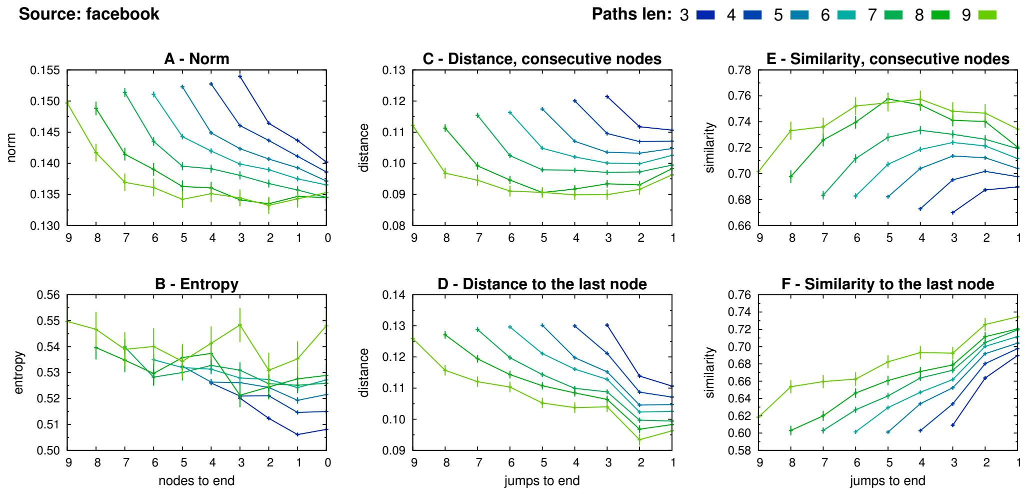

Figure H. Paths generated from the external source *facebook*: averages. As in E.

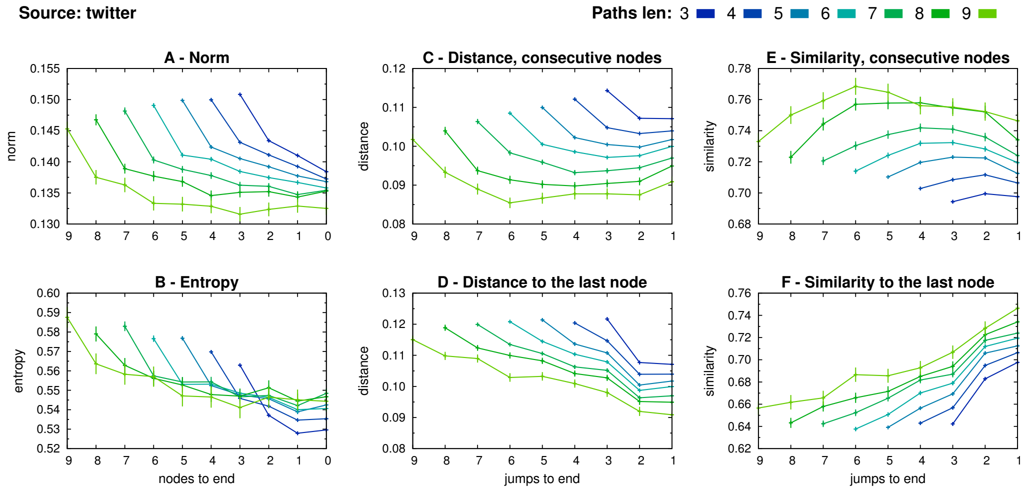

Figure I. Paths generated from the external source *twitter*: averages. As in E.

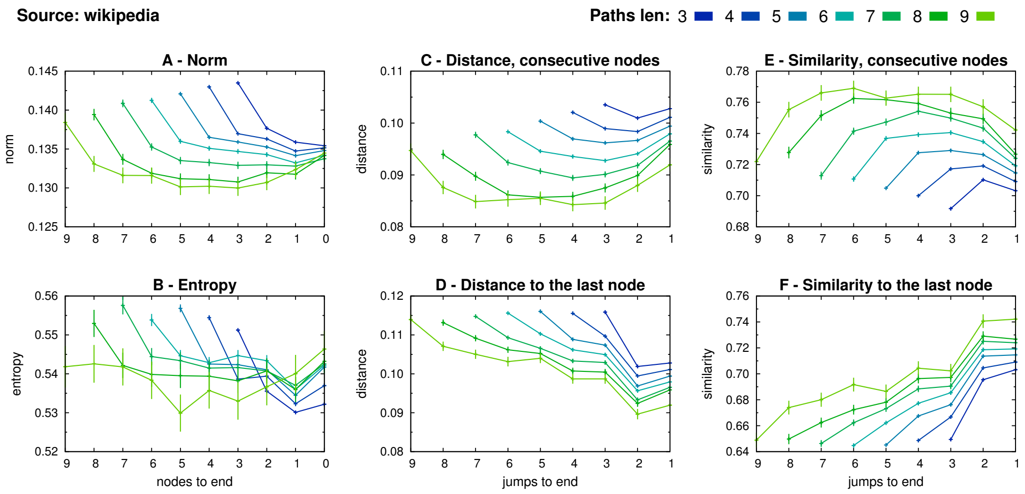

Figure J. Paths generated from the external source *wikipedia*: averages. As in E.

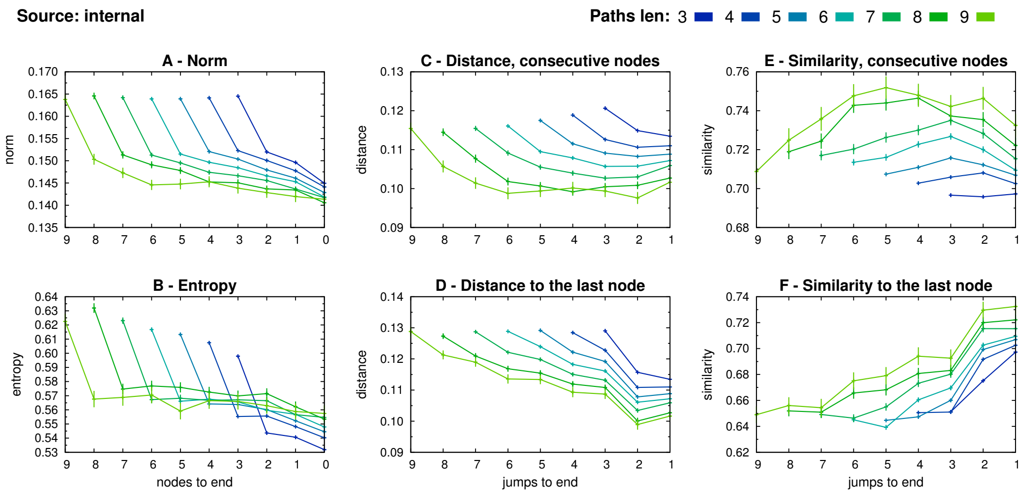

Figure K. Paths generated from the external source *internal*: averages. As in E.

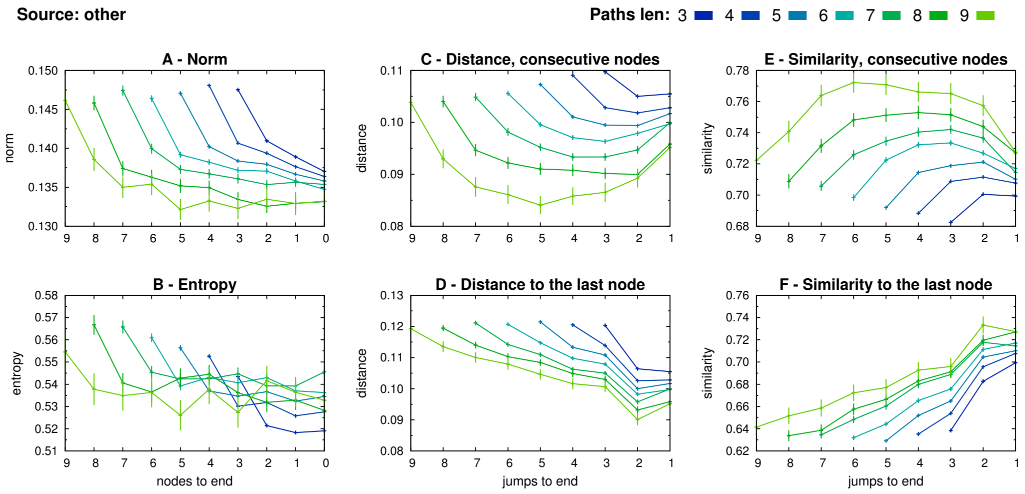

Figure L. Paths generated from the external source *other* (unclassified source): averages. As in E.

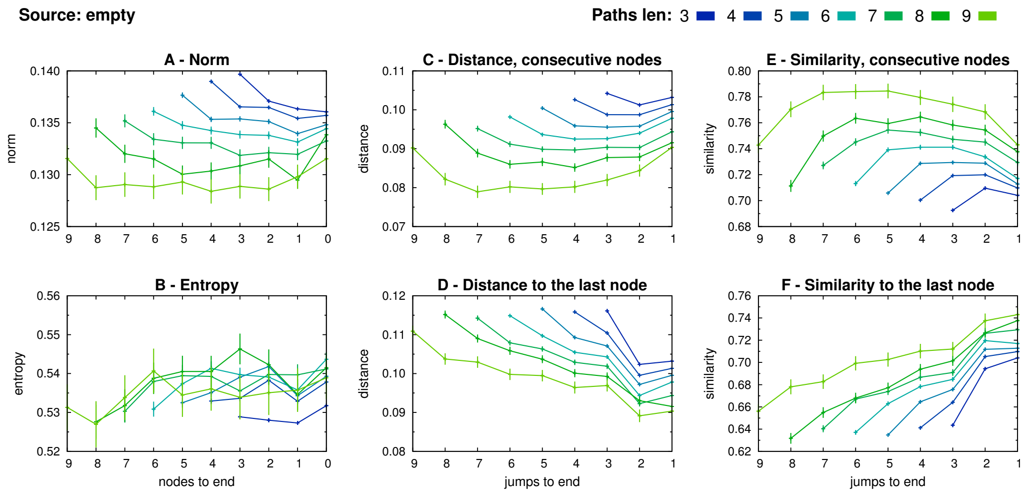

Figure M. Paths generated from the external source *empty*: averages. As in E.
